# Comparison of Nasopharyngeal and Oropharyngeal Swabs for the Diagnosis of Eight Respiratory Viruses by Real-Time Reverse Transcription-PCR Assays

**DOI:** 10.1371/journal.pone.0021610

**Published:** 2011-06-30

**Authors:** Curi Kim, Jamal A. Ahmed, Rachel B. Eidex, Raymond Nyoka, Lilian W. Waiboci, Dean Erdman, Adan Tepo, Abdirahman S. Mahamud, Wamburu Kabura, Margaret Nguhi, Philip Muthoka, Wagacha Burton, Robert F. Breiman, M. Kariuki Njenga, Mark A. Katz

**Affiliations:** 1 U.S. Centers for Disease Control and Prevention, Atlanta, Georgia, United States of America; 2 Centers for Disease Control and Prevention-Kenya, Nairobi, Kenya; 3 Kenya Medical Research Institute, Nairobi, Kenya; 4 International Rescue Committee, Nairobi, Kenya; 5 Ministry of Public Health and Sanitation, Nairobi, Kenya; 6 United Nations High Commissioner for Refugees, Nairobi, Kenya; University of Pretoria/NHLS TAD, South Africa

## Abstract

**Background:**

Many acute respiratory illness surveillance systems collect and test nasopharyngeal (NP) and/or oropharyngeal (OP) swab specimens, yet there are few studies assessing the relative measures of performance for NP versus OP specimens.

**Methods:**

We collected paired NP and OP swabs separately from pediatric and adult patients with influenza-like illness or severe acute respiratory illness at two respiratory surveillance sites in Kenya. The specimens were tested for eight respiratory viruses by real-time reverse transcription-polymerase chain reaction (qRT-PCR). Positivity for a specific virus was defined as detection of viral nucleic acid in either swab.

**Results:**

Of 2,331 paired NP/OP specimens, 1,402 (60.1%) were positive for at least one virus, and 393 (16.9%) were positive for more than one virus. Overall, OP swabs were significantly more sensitive than NP swabs for adenovirus (72.4% vs. 57.6%, p<0.01) and 2009 pandemic influenza A (H1N1) virus (91.2% vs. 70.4%, p<0.01). NP specimens were more sensitive for influenza B virus (83.3% vs. 61.5%, p = 0.02), parainfluenza virus 2 (85.7%, vs. 39.3%, p<0.01), and parainfluenza virus 3 (83.9% vs. 67.4%, p<0.01). The two methods did not differ significantly for human metapneumovirus, influenza A (H3N2) virus, parainfluenza virus 1, or respiratory syncytial virus.

**Conclusions:**

The sensitivities were variable among the eight viruses tested; neither specimen was consistently more effective than the other. For respiratory disease surveillance programs using qRT-PCR that aim to maximize sensitivity for a large number of viruses, collecting combined NP and OP specimens would be the most effective approach.

## Introduction

Acute respiratory illness (ARI) is a significant cause of mortality and morbidity worldwide, especially in young children [1]. Viruses play an important role in ARI, accounting for up to 90% of lower respiratory tract infections in children < 5 years [2].

A variety of sample collection techniques and specimen sources can be used to detect respiratory etiologies, including nasopharyngeal (NP) swabs, oropharyngeal (OP) swabs, nasopharyngeal aspirates (NPAs), nasal swabs, nasal washes, sputa, and saliva specimens. Although NPAs may be the most sensitive specimens, especially when conventional diagnostic methods such as immunofluorescence or culture are used [3], obtaining an NPA is more difficult than obtaining a swab, and collecting NPAs in an outpatient or field setting may not always be feasible [4], [5]. Molecular methods like reverse transcription-polymerase chain reaction (RT-PCR) are becoming widely used for identification of respiratory etiologies [6]. Because molecular tests are more sensitive than conventional methods, less invasive specimen collection techniques than NPA may now approach comparable yields [5], [7].

Depending upon patient characteristics, especially age, obtaining either – or both – NP and OP swabs can be quite physically challenging. Using only one type of swab would be easier logistically, cheaper, and would enable comparisons across surveillance systems. To evaluate the comparative yields of NP and OP swabs in detecting key respiratory viruses by real-time RT-PCR (qRT-PCR), we conducted a prospective study using paired NP and OP specimens from patients at two respiratory disease surveillance sites in Kenya.

## Materials and Methods

### Ethics Statement

Ethical approval for the surveillance activities for influenza and other respiratory viruses was obtained from the Kenya Medical Research Institute (KEMRI) Ethical Review Committee (protocol number 1161). After formal human subjects determination, U.S. Centers for Disease Control and Prevention (CDC) determined this surveillance activity to be nonresearch and therefore approval was not required from the CDC Institutional Review Board. Written informed consent was obtained from adults and from the parents or guardians of minors.

### Study population

The study population consisted of pediatric and adult patients visiting two health-care sites from June 9, 2009 to August 31, 2010, whose illness met the case definition for influenza-like illness (ILI) or severe acute respiratory illness (SARI). The case definitions for ILI and SARI (Table 1) were adapted from those of the World Health Organization [8], [9]. The maximum number of eligible ILI patients was limited to three per day for each site; there was no limit to the number of SARI patients tested. The health-care sites are in the North Eastern and Rift Valley provinces of Kenya and are part of a wider national influenza sentinel surveillance system run jointly by the Kenya Ministry of Public Health and Sanitation and KEMRI/Centers for Disease Control and Prevention-Kenya (CDC-K).

**Table 1:** Case definitions of influenza-like illness and severe acute respiratory infection adapted from World Health Organization^1^^,^ 2

**Influenza-like illness (ILI)**
*All ages (all of the following):*
1. Temperature ≥38°
2. Cough or sore throat
3. Does not meet criteria for SARI
**Severe Acute Respiratory Infection (SARI)**
*For infants ages 1 week to < 2 months (any of the following):*
• Respiratory rate of >60 per minute• Severe chest indrawing• Nasal flaring (when an infant breathes in)• Grunting (when an infant breathes out)• Temperature ≥38°C• Temperature <35.5°C• Pulse oxygenation <90%
*For children ages 2 months to <5 years:*
1. Cough or difficulty breathing
2. AND any one of the following:
• Breathing >50/minute for infant aged 2 months to <1year• Breathing >40/minute for child aged 1 to <5 years• Chest indrawing or stridor in a calm child• Unable to drink or breast feed• Vomits everything• Convulsions• Lethargic or unconscious• Pulse oxygenation <90%
*For persons ages ≥5 years (all of the following):*
1. Temperature ≥38.0°C
2. Cough or sore throat
3. Shortness of breath or difficulty breathing

1. World Health Organization. Handbook: IMCI Integrated Management of Childhood Illness 2005. Available from: http://whqlibdoc.who.int/publications/2005/9241546441.pdf.

2. World Health Organization. WHO Regional Office for Europe guidance for influenza surveillance in humans. 2009. Available from: http://www.euro.who.int/__data/assets/pdf_file/0020/90443/E92738.pdf.

### Specimen collection

NP and OP swabs were separately collected from patients with ILI or SARI by trained surveillance officers. For the NP swab, a polyester-tipped flexible aluminum-shafted applicator (25-801D, Puritan, Guilford, Maine, USA) was inserted into one of the nostrils until resistance was felt at the nasopharynx, then rotated 180 degrees and withdrawn. For the OP swab, a nylon flocked plastic-shafted applicator (503CS01, Copan Diagnostics, Murrieta, CA, USA) was used to sample the posterior oropharyngeal mucosal membrane. After swabbing, the swab applicator was cut off, and each absorbent swab was placed into a vial containing 1 mL of viral transport media (VTM). VTM was prepared at the KEMRI/CDC-K laboratory using standard WHO protocol [10]. Vials were stored at 4°C for up to 72 hours until before shipment to the KEMRI/CDC-K laboratory in Nairobi, where they were stored at −80°C until testing.

### Testing for respiratory viruses

The specimens were vortexed, and a 100-µL volume was used for total nucleic acid extraction using the QIAamp Viral RNA Mini Kit (Qiagen GmbH, Hilden, Germany) according to the manufacturer's instructions. One step qRT-PCR was performed by using the AgPath-ID One-Step RT-PCR Kit (Applied Biosystems, Carlsbad, California, USA). NP and OP specimens from each patient were separately tested by singleplex qRT-PCR for eight viral pathogens: adenovirus, influenza A virus, influenza B virus, human metapneumovirus (hMPV), parainfluenza viruses (PIV) 1–3, and respiratory syncytial virus (RSV). The primers, probes and positive controls for all viruses were provided by CDC-Atlanta. Sequences for the primers and probes are shown in Table 2 [11]. We tested for influenza A virus with the conserved matrix gene-base qRT-PCR; positive influenza A samples were also subtyped as 2009 pandemic influenza A (H1N1) virus (2009 H1N1), influenza A (H3N2) virus (H3N2), and seasonal influenza A (H1N1) virus (H1N1) [12]. Fluorescence was read at the combined annealing-extension step at 57°C and recorded as threshold cycle (C_t_) values. A C_t_ value ≤39.9 was regarded as positive; C_t_ values ≥40.0 were regarded as negative. The qRT-PCR test did not discriminate between viral mRNA and genomic RNA. Specimens were not tested if the following conditions existed when the specimen arrived at the lab: there was no swab, the volume was less than 600 µL, the specimen was at room temperature, patient identification was absent or inadequate, or the patient questionnaire was absent. In addition, the test results were discarded for any specimen whose internal control (human ribonuclease P gene) was negative.

**Table 2:** Primers and probes used in this study.

Assay^1^	Primer/Probe Sequence (5′ to 3′)
Adenovirus	**F** 2 GCC CCA GTG GTC TTA CAT GCA CAT C
	**R** 3 GCC ACG GTG GGG TTT CTA AAC TT
	**P** 4 FAM-TGC ACC AGA CCC GGG CTC AGG TAC TCC GA
hMPV	**F** CAA GTG TGA CAT TGC TGA YCT RAA
	**R** ACT GCC GCA CAA CAT TTA GRA A
	**P** FAM-TGG CYG TYA GCT TCA GTC AAT TCA ACA GA
Influenza A	**F** GAC CRA TCC TGT CAC CTC TGA C
	**R** AGG GCA TTY TGG ACA AAK CGT CTA
	**P** FAM-TGC AGT CCT CGC TCA CTG GGC ACG
Influenza B	**F** TCC TCA ACT CAC TCT TCG AGC G
	**R** CGG TGC TCT TGA CCA AAT TGG
	**P** FAM-CCA ATT CGA GCA GCT GAA ACT GCG GTG
PIV type 1	**F** AGT TGT CAA TGT CTT AAT TCG TAT CAA T
	**R** TCG GCA CCT AAG TAA TTT TGA GTT
	**P** FAM-ATA GGC CAA AGA “T”TG TTG TCG AGA CTA TTC CA^5^
PIV type 2	**F** GCA TTT CCA ATC TAC AGG ACT ATG A
	**R** ACC TCC TGG TAT AGC AGT GAC TGA AC
	**P** FAM-CCA TTT ACC “T”AA GTG ATG GAA TCA ATC GCA AA
PIV type 3	**F** TGG YTC AAT CTC AAC AAC AAC AAG ATT TAA G
	**R** TAC CCG AGA AAT ATT ATT TTG CC
	**P** FAM-CCC ATC TG“T” TGG ACC AGG GAT ATA CTA CAA A
RSV	**F** GGC AAA TAT GGA AAC ATA CGT GAA
	**R** TCT TTT TCT AGG ACA TTG TAY TGA ACA G
	**P** FAM-CTG TGT ATG TGG AGC CTT CGT GAA GCT

1. hMPV, human metapneumovirus; PIV, parainfluenza virus; RSV, respiratory syncytial virus.

2. F, forward primer.

3. R, reverse primer.

4. P, probe.

5. “T”, internal quencher.

### Statistical analysis

Agreement of the results between the paired NP and OP specimens was assessed by using the kappa coefficient. We used the following nomenclature to describe the relative strength of agreement associated with kappa statistics: < 0  =  poor; 0–0.2  =  slight; 0.21–0.4  =  fair; 0.41–0.6  =  moderate; 0.61–0.8  =  substantial; and 0.81–1  =  almost perfect [13]. The assessment was carried out separately for each respiratory virus and for each of the influenza A subtypes.

Similar to previous studies [5], [7], we assessed the sensitivity for each sampling method by considering any positive from either of the specimens as a true positive. We compared the sensitivities using the McNemar's test to account for the correlated binary outcomes from paired data. To avoid bounds above 100%, we used the exact binomial confidence limits. Statistical significance was set at a *p*-value <0.05. Data were analyzed with SAS software version 9.2 (SAS Institute, Cary, NC, USA).

## Results

During the study period, 2,374 paired NP and OP swabs were collected. Forty-three specimens were rejected because of poor quality. Of the 2,331 paired specimens included in the analysis, 754 (32.3%) were from patients with ILI and 1,577 (67.7%) were from patients with SARI. Overall, 1,402 (60.1%) paired specimens were positive for at least one virus, including 393 (16.9%) specimens that were positive for more than one virus. The median number of days from onset of symptoms to specimen collection was 2 days, and 95.9% of samples were collected within 5 days from onset of symptoms (range 0–30 days). The median age of patients was 1 year (range 1 month to 70 years), and 81.6% of patients (n = 1,902) were less than 5 years old (Table 3). Fewer than half (46.1%) of the patients were female (n = 1,074). Adenovirus was the most commonly identified virus, detected in ≥1 specimens from 679 (29.1%) patients (Table 4).

**Table 3:** Demographic characteristics of 2331 patients with influenza-like illness and severe acute respiratory illness from whom paired nasopharyngeal and oropharyngeal swabs were collected – Kenya, 2009–2010.

Category	Subcategory	N (%)
Illness	Severe acute respiratory illness	1577 (67.7%)
	Influenza-like illness	754 (32.3%)
Age	Median	1 year
	Range	1 month–70 years
	<5 years	1902 (81.6%)
	5–17 years	344 (14.8%)
	>18 years and older	85 (3.6%)
Sex	Female	1074 (46.1%)
	Male	1257 (53.9%)
Location	North Eastern Province	1233 (52.9%)
	Rift Valley Province	1098 (47.1%)

**Table 4:** Kappa and sensitivity values of paired nasopharyngeal (NP) swabs and oropharyngeal (OP) swabs for respiratory viruses in all patients (n = 2331) – Kenya, 2009-2010.

Virus^1^	No. positive NP and/or OP swabs	No. positive NP swabs	No. positive OP swabs	% missed with one swab^2^	Kappa statistic^3^ (95% CI)	NP sensitivity (95% CI)	OP sensitivity (95% CI)	*p*-value
Adenovirus	679	391	492	27.5%	0.33 (0.28–0.38)	57.6 (53.8–61.4)	72.4 (68.8–75.7)	< 0.01
hMPV	201	158	139		0.62 (0.55–0.69)	78.6 (72.3–84.1)	69.2 (62.3–75.5)	0.08
Influenza A	256	181	220	14.1%	0.70 (0.64–0.75)	70.7 (64.7–76.2)	85.9 (81.1–90)	< 0.01
2009 H1N1	125	88	114	8.8%	0.75 (0.68–0.82)	70.4 (61.6–78.2)	91.2 (84.8–95.5)	< 0.01
H1N1	6	6	5		0.9 (0.73–1.0)	100 (54.1–100)	83.3 (35.9–99.6)	1.00
H3N2	54	45	50		0.86 (0.79–0.94)	83.3 (70.7–92.1)	92.6 (82.1–97.9)	0.27
unsubtypable	71	33	48		-0.68 (-0.86–0.5)	46.5 (34.5–58.7)	67.6 (55.5–78.2)	0.07
Influenza B	65	55	40	15.4%	0.62 (0.51–0.74)	83.3 (72.1–91.4)	61.5 (48.6 -73.3)	0.02
PIV 1	106	81	71		0.59 (0.50–0.69)	76.4 (67.2–84.1)	67.0 (57.2-75.8)	0.24
PIV 2	56	48	22	14.3%	0.39 (0.25–0.54)	85.7 (73.8–93.6)	39.3 (26.5–53.2)	< 0.01
PIV 3	193	162	130	16.1%	0.66 (0.59–0.72)	83.9 (78.0–88.8)	67.4 (60.3–73.9)	< 0.01
RSV	328	252	247		0.65 (0.59–0.70)	76.8 (71.9–81.3)	75.3 (70.3–79.9)	0.75

1 2009 H1N1, 2009 influenza A pandemic H1N1 virus; H1N1, seasonal influenza A H1N1 virus; H3N2, influenza A H3N2; RSV, respiratory syncytial virus; PIV, parainfluenza virus; hMPV, human metapneumovirus.

2 The percentage of cases that would have been missed if only the more sensitive swab had been used for viruses which had significant differences in sensitivities between swabs.

3 Kappa statistics: < 0  =  poor; 0 – 0.2  =  slight; 0.21 – 0.4  =  fair; 0.41 – 0.6  =  moderate; 0.61 – 0.8  =  substantial; and 0.81 – 1  =  almost perfect agreement. CI, confidence interval.

For all patients combined (ILI and SARI), the relative sensitivities of the swab types varied by virus (Table 4). OP swabs were significantly more sensitive in detecting influenza A virus (85.9% vs. 70.7%, p <0.01), yet sensitivities differed for influenza A subtypes; OP swabs were more sensitive for 2009 H1N1 (91.2% vs. 70.4%, p<0.01), but did not differ from NP swabs in detecting H3N2. OP swabs also were significantly more sensitive than NP swabs for detecting adenovirus (72.4% vs. 57.6%, p<0.01). However, NP swabs had significantly higher sensitivities than OP swabs for influenza B virus (83.3% vs. 61.5%, p = 0.02), PIV 2 (85.7%, vs. 39.3%, p<0.01) and PIV 3 (83.9% vs. 67.4%, p<0.01). The difference in sensitivity for the two types of specimens did not reach statistical significance for hPMV, PIV 1, RSV, H3N2 virus, or unsubtypable influenza A viruses.

For all SARI patients (n = 1,577), OP swabs were significantly more sensitive than NP swabs for adenovirus and 2009 H1N1 virus, but NP swabs were more sensitive than OP swabs for influenza B virus, PIV 2, and PIV 3. For all ILI patients (n = 754), OP swabs were more sensitive than NP swabs for adenovirus and overall influenza A virus; NP swabs were not more sensitive than OP swabs for any of the viruses in this illness category (Table 5). The relative sensitivities of NP and OP swabs from children < 5 years old (n = 1,902) mirrored the overall results. When results in this age group were stratified by SARI and ILI, the comparative sensitivities for NP and OP swabs reached statistical significance in the same pattern as that for all SARI and ILI patients. However, for patients aged 5–17 years (n = 344) and for patients 18 years and older (n = 85), neither swab was significantly more sensitive for any of the viruses, including when results were stratified by SARI and ILI status. For male patients (n = 1,257), specimen performance for all viruses mirrored the overall results. For female patients (n = 1,074), as with the overall results, OP swabs were significantly more sensitive than NP swabs for influenza A virus and adenovirus, while NP swabs were significantly more sensitive than OP swabs for PIV 2; however, there was no statistical difference by swab type for 2009 H1N1 virus, influenza B virus, and PIV 3.

**Table 5:** Kappa and sensitivity values of paired nasopharyngeal (NP) swabs and oropharyngeal (OP) swabs for respiratory viruses by illness category – Kenya, 2009-2010.

Virus^1^	No. positive NP and/or OP swabs	No. positive NP swabs	No. positive OP swabs	% missed with one swab^2^	Kappa statistic^3^ (95% CI)	NP sensitivity (95% CI)	OP sensitivity (95% CI)	*p*-value
**Severe acute respiratory illness (n = 1577)**								
Adenovirus	515	308	367	28.7%	0.32 (0.27–0.38)	59.8 (55.4–64.1)	71.3 (67.1–75.1)	<0.01
hMPV	150	116	101		0.59 (0.51–0.67)	77.3 (69.8–83.8)	67.3 (59.2–74.8)	0.12
Influenza A	168	125	141		0.71 (0.65–0.78)	74.0 (66.7–80.4)	83.9 (77.5–89.1)	0.07
2009 H1N1	77	53	71	7.8%	0.75 (0.66–0.83)	68.8 (57.3–78.9)	92.2 (83.8–97.1)	<0.01
H3N2	41	35	37		0.86 (0.77–0.94)	85.4 (70.8–94.4)	90.2 (76.9–97.3)	0.75
Influenza B	40	35	23	12.5%	0.61 (0.47–0.76)	85.4 (70.8–94.4)	57.5 (40.9–73.0)	0.02
PIV 1	76	57	46		0.51 (0.39–0.63)	75.0 (63.7–84.2)	60.5 (48.6–71.5)	0.15
PIV 2	44	39	15	11.4%	0.36 (0.20–0.53)	88.6 (75.4–96.2)	34.1 (20.5–49.9)	<0.01
PIV 3	155	134	99	13.5%	0.64 (0.57–0.72)	86.5 (80.0–91.4)	63.9 (55.8–71.4)	<0.01
RSV	263	198	198		0.62 (0.56–0.68)	75.3 (69.6–80.4)	75.3 (69.6–80.4)	1.00
**Influenza-like illness (n = 754)**								
Adenovirus	164	83	125	23.8%	0.33 (0.24–0.42)	50.6 (42.7–58.5)	76.2 (69.0–82.5)	<0.01
hMPV	51	42	38		0.71 (0.59–0.82)	82.4 (69.1–91.6)	74.5 (60.4–85.7)	0.52
Influenza A	83	54	74	10.8%	0.68 (0.58–0.77)	65.5 (54.3–75.5)	89.5 (81.1––95.1)	<0.01
2009 H1N1	48	35	43		0.76 (0.65–0.86)	72.9 (58.2–84.7)	89.6 (77.3–96.5)	0.10
H3N2	13	10	13		0.87 (0.72–1.00)	76.9 (46.2–95.0)	100 (75.3–100.0)	0.25
Influenza B	25	20	17		0.64 (0.46–0.82)	80.0 (59.3–93.2)	68.0 (46.5–85.1)	0.58
PIV1	30	24	25		0.77 (0.63–0.90)	80.0 (61.4–92.3)	83.3 (65.3–94.4)	1.00
PIV2	12	9	7		0.49 (0.19–0.80)	75.0 (42.8–94.5)	58.3 (27.7–84.8)	0.73
PIV3	38	28	31		0.70 (0.56–0.84)	73.7 (56.9–86.6)	81.6 (65.7–92.3)	0.63
RSV	65	54	49		0.72 (0.62–0.82)	83.1 (71.7–91.2)	75.4 (63.1–85.2)	0.44

1 2009 H1N1, 2009 influenza A pandemic H1N1 virus; H1N1, seasonal influenza A H1N1 virus; H3N2, influenza A H3N2; RSV, respiratory syncytial virus; PIV, parainfluenza virus; hMPV, human metapneumovirus.

2 The percentage of cases that would have been missed if only the more sensitive swab had been used for viruses which had significant differences in sensitivities between swabs.

3 Kappa statistics: <0  =  poor; 0–0.2  =  slight; 0.21–0.4  =  fair; 0.41–0.6  =  moderate; 0.61–0.8  =  substantial; and 0.81–1  =  almost perfect agreement. CI, confidence interval.

There was a substantial agreement (κ>0.60) between the NP and the OP swabs for all viruses except adenovirus, unsubtypable influenza viruses, PIV 1, and PIV 2 (Table 4). PIV 1 had a moderate strength of agreement (κ = 0.59) between swabs, but adenovirus and PIV 2 had only a fair strength of agreement (κ = 0.33 and 0.39, respectively). Unsubtypable influenza viruses had poor strength of agreement (κ = −0.68). If only the more sensitive swab were used for viruses which had significant differences in sensitivities between swabs, 8.8% –27.5% of cases would have been missed compared to using both swabs. For adenovirus, 72.5% of the total cases detected by using both swabs would have been identified by using the more sensitive OP swab; similarly, 85.9% of all influenza A virus, 91.2% of 2009 H1N1 virus, 84.6% of influenza B virus, 85.7% of PIV 2, and 83.9% of PIV 3 cases would have been detected if only the single, more sensitive swab had been used (Table 4).

## Discussion

To our knowledge, this is the largest study to use qRT-PCR to compare NP and OP swabs for a range of respiratory viruses. We found that the relative performance of specimen type varied by virus. Neither specimen performed uniformly better: NP swabs were more sensitive for some viruses (influenza B virus, PIV 2, and PIV 3), OP swabs were more sensitive for others (overall influenza A virus, 2009 H1N1 virus, and adenovirus), and there was no difference for the rest of the viruses. The large number of patients in this study allowed us to perform comparative statistical analysis with a relatively high degree of precision.

For adenovirus, OP swabs were more sensitive than NP swabs, a finding that was statistically significant in both ILI and SARI patients. This difference may reflect the fact that the major site of initial replication of adenoviruses is the non-ciliated respiratory epithelium of the oropharynx [14]. The kappa value between NP and OP swabs for adenovirus was low (κ = 0.33). This result is consistent with findings of Lambert et al., in which adenovirus accounted for the highest proportion of discordant paired NPA and nasal-throat swab specimens from children [7]. Adenoviruses include over 50 serotypes, and the low concordance between NP and OP specimens for these viruses may reflect different cell tropisms of the adenovirus serotypes for different parts of the respiratory tract [15]. Although we did not conduct serotyping, future studies that evaluate specimen performance for specific adenovirus serotypes could test this hypothesis.

For influenza viruses, sensitivities of NP and OP swabs differed by both type and subtype: NP swabs were more sensitive than OP swabs for influenza B virus, while OP swabs were more sensitive than NP swabs for overall influenza A and 2009 H1N1 virus; there was no significant difference between swabs for H3N2 virus or the unsubtypable influenza A viruses. The sensitivities of NP and OP swabs for unsubtypable influenza A viruses were low, and the strength of agreement between the two swabs was poor. However, unsubtypable influenza A viruses were likely a mix of 2009 H1N1, seasonal H1N1, and H3N2 viruses, making it difficult to interpret this finding. Because half the influenza A specimens were 2009 H1N1, the overall influenza A results were biased towards the 2009 H1N1 findings. Previous studies evaluating NP and OP swabs in detecting influenza viruses found NP swabs to be more sensitive than OP swabs, but these studies used combined outcomes for influenza A and B viruses and did not analyze by influenza A subtypes [6], [16], [17]. The difference in sensitivities of the two swabs in our study may reflect different affinities of influenza types and subtypes for different locations in the respiratory tract. While all influenza viruses infect the respiratory epithelium from the nasopharynx to the bronchioles, 2009 H1N1 virus (and H5N1 virus, which we did not find in our study) can infect lower parts of the respiratory tract, including the alveoli, more commonly than seasonal influenza [18]. This difference could account for the better sensitivity of OP swabs, which reach deeper into the respiratory tract than NP swabs, for 2009 H1N1 virus. Of note, OP swabs have been shown to have superior yield over NP swabs for human cases of avian influenza A (H5N1) [19], [20].

The OP swab sensitivities and kappa values of the parainfluenza viruses were relatively low, with the sensitivity of the OP swab for PIV 2 being the lowest of any virus in our study. This preference for NP swabs is consistent with reports that nasal washes and nasal aspirates have yielded the highest rates of viral recovery for PIV [21].

This study had several limitations. First, we compared only NP and OP swabs; although many routine surveillance systems for influenza and respiratory diseases collect NP and/or OP swabs, other surveillance systems collect NPAs, NP washes, or nasal swabs [22]. While recent studies have used PCR to compare the relative yield of NPAs with those of nose-throat swabs or nasal swabs for respiratory viruses, no studies have evaluated more than three sampling techniques [5], [7], [23], [24]. Head-to-head studies in the future comparing NP swab, OP swab, NPA, NP wash, nose-throat swab, nasal swab, and nasal wash specimen types would provide important information for decisions about which specimens to use for respiratory disease surveillance systems. Second, the NP and OP swabs consisted of different kinds of swab material and used different designs. We used conventional polyester swabs for sampling the nasopharynx and flocked nylon swabs for the oropharynx. Although flocked swabs are superior to conventional swabs for cell recovery, a study comparing different swab material and design (rayon versus nylon flocked swabs) in both the nasopharynx and oropharynx found that the difference in the cycle threshold values between sampling sites was much greater than the difference between swab material and design [25]. Because neither specimen type was consistently more sensitive than the other, we think it is unlikely that the difference in swab material and design substantially affected our results. Additionally, while we tested for eight viruses, we did not include some common viruses, such as coronaviruses and rhinoviruses, and we did not test for bacteria. Finally, the number of adults (n = 85) in this study was relatively small, accounting for just 3.6% of all patients, and as a result there was limited power to compare the sensitivities of NP and OP swabs for specific viruses in this population.

In summary, NP and OP specimens collected from patients with respiratory illness had variable sensitivities by qRT-PCR for eight viruses. Neither specimen was consistently more sensitive than the other. Collecting both swabs had a complementary effect; even when there was higher sensitivity for one technique over the other, the lower-sensitivity technique still identified a considerable number of cases not identified by the higher-sensitivity one. For respiratory disease surveillance programs using qRT-PCR that aim to maximize sensitivity for a large number of viruses, collecting combined NP and OP specimens would be the ideal approach. However, the enhanced sensitivity of using both swabs comes at a higher cost; this includes not only the expense of the second swab, but further patient discomfort as well as additional time and effort from the person taking the sample. Thus, for surveillance systems with limited resources, a single-swab approach, whether NP or OP, would be the most logistically simple and maintain moderate sensitivity for many of the pathogens we tested in our study.
